# Tuning synaptic strength by regulation of AMPA glutamate receptor localization

**DOI:** 10.1002/bies.202400006

**Published:** 2024-05-01

**Authors:** Imogen Stockwell, Jake F. Watson, Ingo H. Greger

**Affiliations:** 1 Neurobiology Division, MRC Laboratory of Molecular Biology, Cambridge, UK; 2 Institute of Science and Technology, Technology (IST) Austria, Klosterneuburg, Austria

**Keywords:** AMPA receptor traffic, AMPA receptor structure, cryo-EM, long-term potentiation, synaptic plasticity, short-term plasticity

## Abstract

Long-term potentiation (LTP) of excitatory synapses is a leading model to explain the concept of information storage in the brain. Multiple mechanisms contribute to LTP, but central amongst them is an increased sensitivity of the postsynaptic membrane to neurotransmitter release. This sensitivity is predominantly determined by the abundance and localization of AMPA-type glutamate receptors (AMPARs). A combination of AMPAR structural data, super-resolution imaging of excitatory synapses, and an abundance of electrophysiological studies are providing an ever-clearer picture of how AMPARs are recruited and organized at synaptic junctions. Here, we review the latest insights into this process, and discuss how both cytoplasmic and extracellular receptor elements cooperate to tune the AMPAR response at the hippocampal CA1 synapse.

## Introduction

Understanding how routes of neuronal communication are stored and later recalled has been a longstanding quest in neuroscience research. Synaptic plasticity, where connections between specific neurons are altered in response to ongoing activity, is thought to underlie much of this memory storage. Since the initial discovery of long-term potentiation (LTP) in the hippocampus,^[1]^ the mechanisms controlling the strength of synaptic transmission have been intensely investigated. Two main mechanisms for this phenomenon have been proposed: a presynaptic change in L-glutamate release, and a change in the postsynaptic sensitivity to this neurotransmitter, which is mediated by the fast-acting ionotropic glutamate receptors (iGluRs).^[2]^ The realization that the AMPAR response can selectively increase during LTP,^[3–6]^ together with the demonstration that this increase can be triggered in the absence of a presynaptic terminal,^[7]^ has shifted the study of AMPAR synaptic regulation into the limelight. Outlining the molecular dissection of AMPARs that has since followed in an attempt to locate these regulatory mechanisms will be the focus of this review (see also ^[8]^).

**Figure 1:**
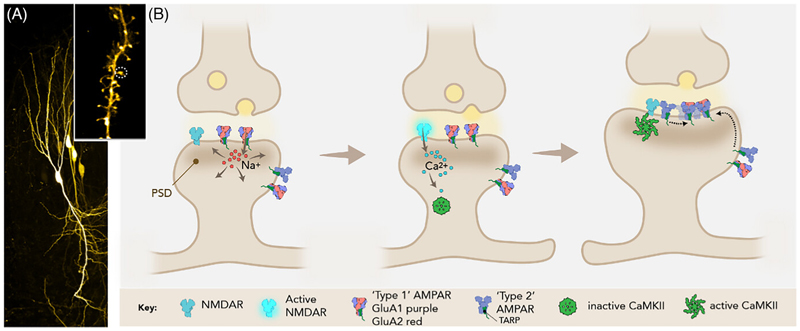
(A) Confocal image of GFP-expressing hippocampal pyramidal neurons in an organotypic hippocampal slice. Inset shows a zoomed in region of dendrite, imaged by STED microscopy, with a postsynaptic spine circled in white. **(B)** Schematic of the postsynaptic molecular changes occurring during potentiation of an excitatory glutamatergic synapse. Glutamate release from presynaptic vesicles activates postsynaptic AMPARs, which enable the influx of Na^+^ ions to depolarize the postsynaptic cell (panel 1), thereby activating NMDARs and the influx of Ca^2+^ ions (panel 2). Subsequent postsynaptic Ca^2+^ signaling processes, such as the activation of CAMKII (panel 3), result in the recruitment of further AMPARs and their organization into transsynaptic nanocolumns, as well as the growth of the postsynaptic density and protrusion of the dendritic spine.

AMPARs are a class of iGluRs: a family of glutamate-gated cation channels that also includes NMDA, kainate, and delta (GluD) receptors.^[9]^ AMPARs and NMDARs are most widely expressed at excitatory synapses, where AMPARs mediate the majority of fast excitatory transmission, providing the initial postsynaptic depolarization that is essential for subsequent NMDAR activation; synapses lacking AMPAR are functionally silent.^[4,5]^ At hippocampal Schaffer collateral-CA1 synapses, NMDAR activation allows the influx of Ca^2+^ ions, which in turn trigger downstream signaling processes, culminating in the expression of LTP through the recruitment and subsynaptic organization of AMPARs (Figure 1).

## Ampars at Excitatory Synapses

Excitatory synapses are predominantly formed on dendritic spines, which are micron-sized membrane protrusions that make up the postsynaptic element opposing presynaptic axon terminals (Figure 1A). Spines are supported by a cytoskeletal framework enriched in filamentous (F)-actin, and harbor the postsynaptic density (PSD): a complex network of structural and signaling proteins that is aligned with presynaptic vesicle release sites.^[10,11]^ AMPARs diffuse in the plane of the postsynapse ^[12]^ and connect to the MAGUK (membrane-associated guanylate kinase) family of PDZ-containing proteins, either directly via their C-terminal PDZ-binding motifs,^[13,14]^ or indirectly via the C-tails of transmembrane AMPAR-associated auxiliary proteins (TARPs).^[15,16]^ Interaction of the TARP cytosolic C-tail with PSD-93/95, the most abundant MAGUKs in the PSD, is a major mechanism of AMPAR synaptic recruitment.^[17]^ This cytoplasmic anchor-age physically links receptors to the PSD, limiting their diffusion away from the critical sites for synaptic transmission.^[18–21]^ Not only does the PSD capture and retain AMPARs in this manner, but is also able to organize them.^[22]^ Superresolution imaging has demonstrated subsynaptic organization of MAGUKs, which can concentrate subsynaptic populations of AMPARs.^[19,23,24]^ Optimizing the alignment of these clustered AMPARs with presynaptic neurotransmitter release sites may provide a mechanism for the control of synaptic strength.^[24–26]^ A central player upstream of AMPAR recruitment is calcium calmodulin-dependent kinase II (CaMKII), which is activated by Ca^2+^ influx through NMDARs, upon which it translocates to the PSD (Figure 1B).^[27]^ CAMKII is involved in the synaptic immobilization of AMPARs.^[28]^ However, how CAMKII facilitates the recruitment of additional AMPARs during LTP is not established; this central issue remains a matter of ongoing debate.^[29]^

On the extracellular side, AMPARs face the synaptic cleft, an environment densely packed with various transsynaptic factors, including adhesion proteins, secreted synaptic organizers, and the extracellular matrix.^[26]^ Whilst the cytoplasmic influence on AMPAR localization has been intensively studied for decades, only recently has the importance of the AMPAR extracellular domain in LTP been dissected.^[30,31]^ Together, this dual setting of cleft and cytosol will determine the activity-dependent AMPAR subsynaptic organization.^[26,32]^ Here, we review our current understanding of the contribution of cytosolic interactions of AMPAR-TARP complexes with the PSD on the one hand, and of receptor extracellular components in the synaptic cleft on the other.

## Ampar Organisation

Like all iGluRs, AMPARs exist in the PSD as tetramers, assembled from four core subunits, GluA1-4, in various combinations.^[33]^ Inclusion of the GluA2 subunit renders the tetramer impermeable to Ca^2+^ ions, and these “Type-1” AMPARs predominate in pyramidal neurons across the forebrain. GluA2-lacking, “Type-2” receptors on the other hand are Ca^2+^ permeable. They are abundantly expressed in interneurons and in glia, but are rare in pyramidal neurons, where the GluA1 homotetramer is thought to be the most prominent variety.^[34]^ Various lines of evidence suggest that GluA1 homomers in CA1 pyramidal neurons can be induced transiently by LTP stimuli, with their Ca^2+^ signal contributing to the expression of LTP ^[35–37]^ (but see ^[38]^). Type-2, Ca^2+^-permeable receptors would be subject to different regulatory trafficking mechanisms, due not only to sequence diversity in their C-tails and N terminal domains (NTDs), but also as a result of their distinct NTD structure ^[39]^ (see below).

**Figure 2:**
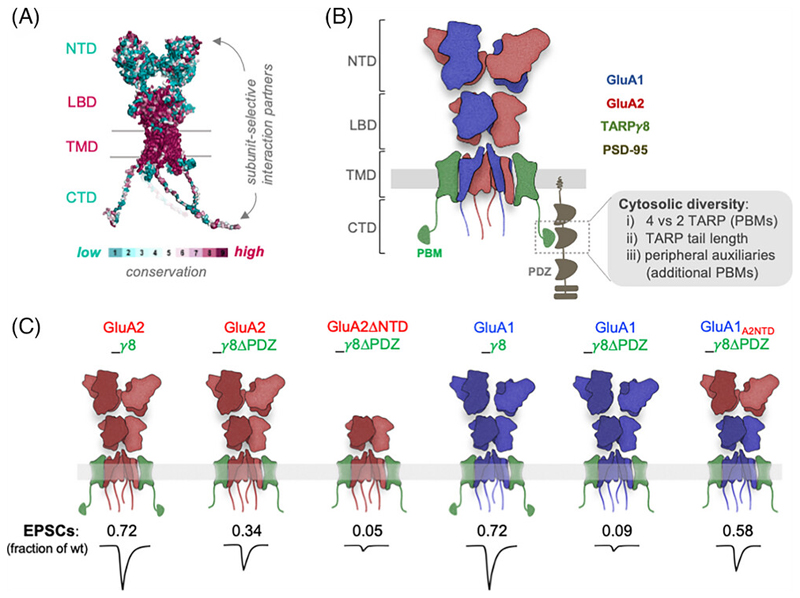
(A) AMPAR structure colored by sequence conservation between the four subunits (NTD: N terminal domain, LBD: ligand-binding domain, TMD: transmembrane domain, CTD: C terminal domain). The LBD and TMD sequences are highly conserved (magenta), whilst the NTD and CTD show greater sequence diversity (cyan), enabling subunit-specific interactions. **(B)** Schematic of a heteromeric AMPAR on the postsynaptic membrane, held in the postsynaptic density by interactions between the associated auxiliary protein TARP*γ*8 and the three PDZ domains of PSD95, and the NTD that extends into the synaptic cleft. Cytosolic diversity of synaptic AMPAR complexes can arise by (i) the stoichiometry of associated TARPs (ii) TARP type, for example, different length C-tails of TARP *γ*2 and TARP*γ*8 (iii) PDZ anchoring by additional associated proteins, for example, CKAMPs. **(C)** Schematic depicting the subunit-specific effect of the NTD and TARP*γ*8 PDZ interactions on synaptic transmission. Excitatory postsynaptic current (EPSC) amplitude, normalized to a neighboring transfected neuron, is reduced when removing the TARP*γ*8 PDZ binding motif (*γ*8ΔPDZ). *γ*8 PDZ deletion prevents GluA1-mediated synaptic transmission, which can be partially rescued by the NTD of GluA2 (derived from Watson et al., 2021).

AMPARs share the modular design of other iGluRs and are composed of four distinct domains: the extracellular NTD and ligand binding domain (LBD; binding the agonist L-glutamate), the transmembrane domain (TMD) forming the ion channel, and the unstructured intracellular C terminal domain (CTD) ^[9,40]^ (Figure 2A,B). The NTD and LBD form dimers of dimers, whilst the TMD is four-fold symmetric.^[41]^ Contrary to the evolutionarily conserved LBD and TMD between the four AMPAR subunits, the NTD and CTD are the most sequence-diverse regions (Figure 2A), and therefore enable subunit-selective protein interactions and functions. The NTD encodes roughly 50 percent of an AMPAR subunit (~ 400 amino acids), whilst the CTD is only 50–80 amino acids in length.^[13,42]^ Unique to AMPARs amongst iGluRs is the diversity of auxiliary subunits that they associate with. The type and expression level of auxiliary subunits varies by brain region, with consequences for gating and trafficking.^[9,16,40,43]^ Both type and stoichiometry of auxiliary subunits on a given AMPAR will also significantly impact synaptic anchoring, as some auxiliaries interact with the PSD scaffold, while others do not.

## Role of Cytoplasmic Elements in Synaptic Recruitment

Initial studies of AMPAR synaptic trafficking and anchorage focused on cytoplasmic CTD interactions as the primary mechanism. This work was inspired by the observation that NMDARs are linked to PSD-95 through their extreme C-termini,^[44]^ and what followed was extensive cloning of various multi-PDZ domain-containing proteins which interact with AMPARs.^[13,14]^ Moreover, subunit differences in CTD sequence, together with the fact that GluA1 ^[45]^ but not GluA2 ^[46]^ is essential for various forms of LTP, focused further attention on the CTD. Hippocampal CA1 pyramidal neurons mainly express the GluA1 and GluA2 subunits, and to a lesser extent GluA3.^[47]^ Interestingly, these three subunits are subject to different trafficking mechanisms, with GluA1 selectively accumulating at the cell surface to form an extra-synaptic “reserve” pool ^[47]^ that is critical to supply AMPARs during LTP.^[48,49]^ Overexpression studies demonstrated distinct subunit-specific synaptic trafficking profiles, where GluA2 homomers (and GluA2/GluA3 heteromers) were shown to traffic into the synapse constitutively,^[50]^ whilst the synaptic insertion of GluA1 required either LTP stimuli, constitutively active CAMKII,^[50,51]^ or overexpressed PSD-95.^[52,53]^ These differences were ascribed to the CTD, its cytoplasmic interactors and to subunit-selective CTD phosphorylation.^[54]^ However, mutating the CTD protein interaction sites,^[48,55]^ or even complete CTD removal, does not prevent synaptic targeting of GluA1 or the expression of LTP ^[48,56]^ (but see ^[57]^). Therefore, subunit-specific trafficking as a principle has been established, but is not solely orchestrated by their CTDs.

An additional cytosolic role is played by AMPAR auxiliary subunits, primarily the six-membered TARP family,^[17,58]^ which centrally regulate AMPAR trafficking and synaptic anchoring.^[16,40,43]^ TARPs direct AMPARs both from the endoplasmic reticulum to the cell surface, and from the surface into the synapse, where they impact receptor stability.^[17,21,59]^ A PDZ-binding motif (PBM) on the extreme C-tails of Type-1 TARPs (TTPV-COOH) connects AMPARs to PSD-93/95 ^[60–62]^ (Figure 2B). Deletion of the TARP-*γ*2 C-tail or of the TARP-*γ*8 PBM results in a more diffuse surface distribution of receptors,^[55,61]^ highlighting the importance of TARP-PSD95 interactions in accumulating AMPARs at synaptic sites. Other than TARPs, CNIH (cornichon homolog) auxiliary subunits are strongly expressed in the hippocampus.^[63,64]^ These proteins recognize the same four binding sites on the AMPAR TMD as the TARPs do,^[65]^ but their C-terminus is extracellular and lacks a PBM.^[65]^ Therefore, the type and stoichiometry of auxiliary subunits will determine the number of PSD-interacting anchors of a given AMPAR complex (Figure 2B). In hippocampal pyramidal neurons, the predominant AMPAR is the GluA1/2 heteromer,^[47]^ which associates with two TARP and two CNIH subunits.^[66,67]^ However, other minor receptor subtypes that are associated with different sets of auxiliaries likely exist in these neurons, and are expected to have very different functional,^[68]^ trafficking, and anchoring properties.^[43]^ Adding further complexity is the presence of peripheral auxiliary sub-units of the CKAMP/Shisha family.^[69,70]^ These also harbour PBMs in their C-termini,^[71]^ but their stoichiometry and mode of association with the AMPAR are currently elusive.

Given the prominent role of CaMKII in LTP, the regulatory effects of phosphorylation of the CTDs of both AMPARs and TARPs has been investigated as a possible downstream target for CaMKII.^[27,29,54]^ In the case of TARPs, CTD phosphorylation has been suggested to cause their dissociation from negatively charged lipids of the inner membrane leaflet.^[72]^ Release of the TARP tails from the membrane by charge repulsion would effectively increase their reach for the PDZ domains of PSD-95.^[73]^ In addition, preventing the phosphorylation or dephosphorylation of TARP-*γ*2 blocked both LTP and LTD respectively.^[74]^ LTP is also reduced (by 60%) in a phosphor-null mutant TARP-*γ*8 knockin mouse.^[75]^ However, more recent experiments suggest that phosphorylation of the TARP tails reduces their binding to multiple sites on PSD-95, in addition to the canonical PDZ binding motif.^[76]^ Moreover, in a study focusing on GluA1 homomers, the TARP-*γ*8 phospho-null mutant did not affect LTP,^[77]^ suggesting that in some AMPAR/TARP complexes TARP phosphorylation has a non-essential role. Taken together, the mechanisms linking CaMKII activation to AMPAR synaptic insertion remain to be elucidated.

In addition to acting as a kinase, CaMKII has been shown to organize proteins by liquid-liquid phase separation, with Ca^2+^ triggering the segregation of PSD-95 and TARP-*γ*2 into a central condensate, thereby separating AMPAR and NMDAR nanoclusters.^[78]^ The entire C-tail of TARP-*γ*2 has been shown to undergo phase separation with PSD-95 in vitro, with tail regions other than the PBM engaging PSD-95 and contributing to synaptic clustering.^[76]^ An increase in Ca^2+^ concentration restricts the diffusion of AMPARs on the membrane,^[79]^ likely a combined effect of the dual roles of CaMKII phosphorylation of TARP-*γ*2 trapping AMPARs at the synapse and causing phase separation.^[28]^ Together, these mechanisms enrich the binding capacity of synaptic sites, thereby enhancing AMPAR recruitment and synaptic transmission.^[80]^

## Subunit-Specific Requirement for Ntd Versus Tarp-Mediated Anchoring

Despite the nonselective interaction between TARPs and AMPAR subunits, there are nevertheless subunit-specific dependencies on TARP interactions. The TARP—PSD-95 anchor is of greater significance for GluA1 than GluA2, as seen using ‘tandem’ constructs, where the TARP is fused to the GluA C-tail (via the TARP N-terminus).^[81]^ This setting allows control of both AMPAR subunit, and TARP-PDZ interactions. Deletion of the TARP-*γ*8 PBM in a GluA1-TARP-*γ*8 tandem (GluA1_*γ*8ΔPDZ) fails to rescue excitatory postsynaptic currents (EPSCs) when expressed in an AMPAR null genetic background ^[55,77]^ (Figure 2C). Interestingly, GluA2 is less sensitive to TARP PBM deletion, as the GluA2_*γ*8ΔPDZ receptor construct can partially rescue AMPAR synaptic currents. This effect is mediated by the GluA2 NTD, which also appears to have a strong affinity for synaptic sites, and when placed on GluA1, can facilitate synaptic anchoring in the absence of TARP interactions. Lastly, in a GluA1/GluA2 heteromeric receptor with both subunits lacking the TARP PBM (*γ*8ΔPDZ), the NTD of the GluA2 subunits is also capable of rescuing synaptic transmission.^[55]^ These results add to our picture of subunit-specific recruitment: GluA2-lacking receptors are more dependent on TARPs than GluA2-containing receptors due to the dominating synaptic recruitment ability of the GluA2 NTD ^[55]^ (Figure 2C).

## Role of the Ampar Ntd in Synaptic Recruitment

A role for the AMPAR NTD in synaptic anchoring was first suggested for the GluA4 subunit at interneuron synapses.^[82–84]^ However only recently has the role of the NTD been fully appreciated at principal neuron synapses, with subunit specific effects on LTP.^[30,31,55,56,85]^ It appears that historically used AMPAR N-terminal GFP tags,^[50,86]^ occlude the contribution of GluA1 to synaptic transmission,^[30,31]^ and therefore overestimated the role of CTD interactions in AMPAR targeting. The GFP tag may cause steric hindrance to the synaptic entry of GluA1, but also prevent contact with NTD interacting proteins critical for synaptic anchorage of the receptor. Of interest, an N-terminal GFP on GluA2 is less detrimental to synaptic transmission than on GluA1, with tagged GluA2 still entering the synapse readily.^[30,31]^ The role of the NTD in AMPAR anchoring was further confirmed using NTD-deleted receptors, which have subunit-specific impairments in transmission. In particular, maintenance of LTP was prevented by GluA1 NTD deletion, but not GluA2.^[30,31,55,85]^ The subunit-specific roles of NTD interactions, with GluA1 implicated in LTP and GluA2 for basal transmission levels, echo subunit trafficking rules ascribed to CTDs, yet how the NTD manifests these effects has not yet been resolved.^[8]^ It should be noted that while the above experiments using receptor overexpression reported a clear difference between the GluA1 and GluA2 NTD in synaptic anchoring, GFP-tagged GluA1 knock-in mice do display normal receptor trafficking,^[87,88]^ suggesting that the GFP appendage is not an absolute block to receptor localization under different experimental conditions.

**Figure 3:**
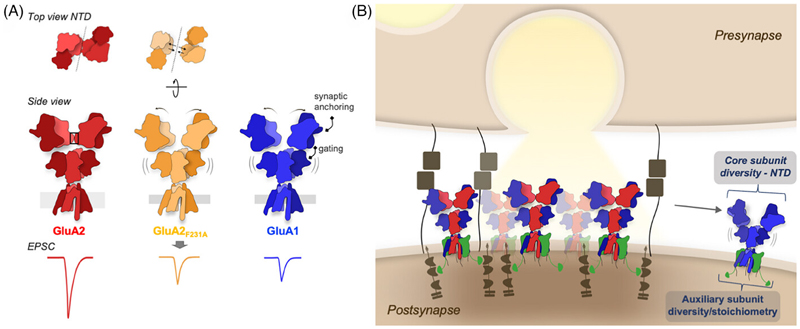
(A) Proposed model of the role of the NTD in synaptic anchoring of AMPARs. The stability of the tetrameric interface in GluA2-containing receptors (highlighted in black; ‘side view’) may enable efficient synaptic anchoring, as receptors without this interface (GluA1 homomers) or with a disrupted interface (GluA2_F231A_) show reduced EPSCs following Schaffer collateral stimulation. The flexibility of the NTD with a disrupted interface has implications for both the gating and synaptic anchoring of the receptor. **(B)** Synaptic accumulation of AMPARs is maintained by TARP (green) PDZ interactions with PSD-95 (brown). Subsynaptic positioning into receptor clusters opposing vesicle release is influenced by both the core subunit, determining subunit-specific NTD interactions, and the associated auxiliary proteins providing PSD-95 anchoring. NTD interactions with synaptic cleft molecules may be disturbed in receptors with a broken NTD dimeric interface, making them less likely to be maintained in a stable synaptic position.

The function of the NTD in the regulation of synaptic transmission may be multi-layered. This domain is not simply a static platform for interactions, but can be highly mobile.^[42]^ Structural studies,^[89–91]^ complemented by simulations ^[92,93]^ have demonstrated large motions of NTD dimers accompanying receptor gating, and an effect of domain removal on channel kinetics has been reported.^[94]^ A recent atomic-force microscopy study even suggests NTD dimer splitting into monomers,^[95]^ which is surprising given the low nanomolar interaction between NTD monomers,^[33,96,97]^ but may permit clustering between adjacent receptors through NTD monomer interactions in *trans*.^[95]^ Glutamate-induced receptor gating motions would have the potential to alter synaptic interactions, but this may be reduced in the context of a crowded synaptic cleft environment. Recent structural data has developed these ideas to demonstrate that the effect of NTD motions are also subunit-specific. Cryo-Electron Microscopy structures revealed that the NTD tier of GluA1 homomers is uniquely flexible compared to GluA2-containing receptors.^[39]^ In GluA2, an interface between NTD dimers holds receptors in a compact “Y” shaped structure ^[41]^ (Figure 3A). This interface is absent in GluA1 (due to NTD sequence divergence), resulting in a wide spectrum of GluA1 NTD dimer configurations. Destabilizing the GluA2 NTD interface with a point mutation (F231A) renders the GluA2 NTDs equally flexible (Figure 3A).^[39]^ Unlike unmodified GluA2 receptors,^[30,31]^ the GluA2Q_F231A_ mutant fails to boost EPSCs in CA1 pyramidal neurons, thus closely mimicking GluA1 ^[39]^ (Figure 3A, bottom). How NTD dynamics affect AMPAR synaptic recruitment in LTP remains to be understood. The critical GluA2 NTD interface is important for more than just GluA2 homomers: the preferred building plan of heteromeric receptors (GluA1/2 and GluA2/3) places GluA2 subunits in position to maintain this interface in the majority of GluA2-containing receptors (Figure 2B).^[98,99]^ Taken together, this supports the idea that the NTD may provide an “anchoring platform” that can adopt state and subunit-dependent conformations that affect receptor anchoring, and in turn impact various forms of synaptic plasticity (Figure 3).

Based on these data, we propose that the constitutive recruitment of GluA2-containing AMPARs requires a compact, tetrameric sNTD platform, enabled by a GluA2-specific NTD interface (Figure 3A, top). Structural integrity of this platform determines interactions with anchoring proteins, and these interactions are altered or occluded by flexible NTD motions, occurring in GluA1, GluA2_F231A_, and in particular, desensitized receptor conformations.^[39]^ This model explains both the strong ‘synapto-sticky’ phenotype of the GluA2 NTD, which can support synaptic anchoring alone, and the decreased synaptic anchoring of GluA1 and GluA2_F231A_.^[31,39]^ The necessity of the GluA1 NTD for LTP suggests a role for specific interactors, which could act by stabilizing an otherwise mobile NTD tier. How NTDs contribute to subunit-selective delivery of heteromeric receptors is unclear. Both GluA1/GluA2 and GluA2/GluA3 heteromers are expected to exhibit a compact (tetrameric) NTD mediated by the GluA2-specific NTD interface (Figure 2B), leaving the GluA1 or GluA3 subunits to occupy the more exposed outer position within the tetramer. This has the potential to enable subunit-specific interactions and trafficking rules, which remain to be fully clarified.

## Ntd Interactors in the Synaptic Cleft

The molecules directing AMPAR function through the NTD could extend from either pre or postsynapse, or be secreted factors. More generally, the mesh of proteoglycans and glycoproteins that makes up the extracellular matrix can constrain the diffusion of AMPARs but not NMDARs, affecting short term plasticity by limiting the exchange of AMPARs within the synapse.^[100]^ Indeed, it is of interest that the synaptic occlusion of GluA1-GFP is not observed in dissociated neuronal culture lacking such an environment.^[86]^ AMPARs uniquely form macromolecular complexes with numerous synaptic proteins,^[42,69,101,102]^ amongst which the role of secreted neuronal pentraxins is the best studied. As pointed out above, these were the first NTD-interacting proteins described and have a capacity to cluster receptors within the synaptic cleft.^[88]^ Pentraxins preferentially bind the NTD of GluA4 and act as a transsynaptic organizer in interneurons.^[82,83]^ At excitatory synapses, the search for synaptic cleft interactions that could capture and retain AMPARs is still ongoing. A recent study has characterized neuroplastin-65, a single transmembrane postsynaptic cell adhesion molecule, showing it to interact specifically with the NTD of GluA1 and to be required for the maintenance of LTP,^[85]^ but further work is required to understand how this anchor is employed at the synapse. Moreover, members of the Noelin/Olfactomedin family interact with both the AMPAR extracellular region and various extracellular proteins ^[103]^ to form a network of molecules potentially linking pre and postsynaptic neurons. Strong effects on both synaptic transmission and plasticity observed in Noelin knockout mice suggest a model whereby extracellular factors are required for the stable trapping of AMPARs at both the cell surface and synapse.^[104]^ Transsynaptic receptor interactions are not just proposed for AMPARs; such interactions appear to be an organizing principle utilized across synapse types. For example, Cbln1, released from cerebellar granule cells, links presynaptic neurexins and the NTD of postsynaptic GluD2 to form a transsynaptic organizer complex.^[105–107]^ Similarly, C1q-like secreted proteins bridge post-synaptic kainate receptors to presynaptic neurexin.^[108]^ Given the dense protein network of the synaptic cleft, understanding receptor organization in their native context is becoming increasingly important to fully appreciate the mechanisms controlling synaptic transmission.

## Transsynaptic Nanocolumns

Advances in super-resolution light microscopy has illuminated the subsynaptic organization of AMPARs. Both PALM and STORM imaging revealed the existence of AMPAR nanodomains of ~70–80 nm in diameter, containing 20–25 receptors per cluster.^[19,23,24,109]^ Other iGluRs are also not homogeneously distributed at the synaptic membrane,^[110]^ and are specifically localized for their signaling functions.^[111]^ AMPARs have a relatively low affinity for glutamate, so clustering of receptors can efficiently increase synaptic currents without necessitating increased receptor production or trafficking.^[112–114]^ Simulations suggest that the displacement of AMPAR clusters by at least 100 nm would result in a reduction in EPSC amplitude,^[19]^ therefore a transsynaptic alignment of AMPARs will determine postsynaptic current amplitudes. Indeed presynaptic proteins, such as voltage-gated calcium channels and vesicle priming molecules required for transmitter release, for example, RIM and Munc-13, are similarly clustered.^[26,115,116]^ Alignment of pre and postsynaptic nanoclusters into a transsynaptic “nanocolumn” ^[24,26,116]^ would enable efficient activation of postsynaptic receptors, and provide a mechanism for tuning the strength of transmission in addition to simply increasing the number of synaptic receptors. Yet, if and how these mechanisms occur and contribute across the timescales of synaptic plasticity remains to be answered. It should also be emphasized that AMPAR nanocolumns are not ubiquitously observed across excitatory synapses; they have not been seen in high-throughput synapses (that are tuned to coincidence detection),^[117]^ and may be a hallmark for synapses optimized to integrate presynaptic signals.

Alignment of receptors with presynaptic release sites likely requires transsynaptic interactions. One recently identified interactor is LRRTM2, cleavage of which induces the dispersal of AMPARs from RIM1/2-labelled release sites.^[118]^ LRRTMs plug into the PSD, so could align AMPARs to the presynapse indirectly, through TARP/PSD-95 linkage, or organize receptors through direct interactions.^[69]^ The AMPAR NTD, projecting halfway into the synaptic cleft and reporting the conformational state of the receptor, would provide a prime anchor to link pre and post synapse. Whilst NTD deletion does not result in the diffuse surface distribution of receptors seen upon TARP-*γ*8 PBM deletion, some changes in the subsynaptic distribution of NTD-deleted receptors can be observed.^[55]^ As PSD-95 appears to align with presynaptic vesicle release,^[23,24]^ TARP interactions may be sufficient for the formation of AMPAR nanocolumns,^[55]^ yet the role of each AMPAR interactor for anchoring, clustering, and alignment of the receptor requires careful further investigation.

## Ampar Anchoring and Short-Term Plasticity

The interplay between AMPAR lateral diffusion by Brownian motion ^[20,22]^ and their anchoring within trans-synaptic nanocolumns is not only relevant for the expression of LTP,^[48,49,119]^ but is also expected to impact short-term plasticity (STP).^[79]^ The rate of AMPAR diffusion appears to be influenced by their conformation, with desensitized receptors showing an increased mobility.^[79]^ This mobility had previously been linked to decreased TARP association,^[120]^ however recent experiments demonstrate no effect of glutamate on TARP association in functional ^[121,122]^ and structural studies.^[39,123–125]^ Desensitization-induced rearrangement of the NTD tier, followed by detachment from an anchoring protein is a possible alternative mechanism (Figure 3B), and may contribute to STP by altering paired-pulse facilitation.^[39]^ These results suggest that an interplay between receptor desensitization kinetics and receptor diffusion is dictating STP, due to the need to stably position receptors opposing glutamate release. Subunit-specific NTD interactions likely contribute to these behaviors and thereby impact both STP and LTP.^[126]^

## Conclusions and Outlook

The view that memory involves rapid and long-term changes in the strength of synaptic transmission is longstanding, persisting since Hebb’s 1949 postulation that memory formation involved “organization by structural modifications.” The current model of synaptic function is highly dynamic: receptor diffusion, phase separation and transsynaptic interactions cooperatively function to provide a modifiable channel of communication. With dynamic reconstruction occurring at both the presynaptic active zone and postsynaptic density, it is the alignment of receptors to glutamate release that can dramatically alter transmission strength.^[26]^ As discussed, diverse AMPAR populations will have unique conformational landscapes, with the dynamics of receptor diffusion in the synaptic cleft being tuned by protein interactions. Linking the molecular architecture of the synapse to the long-term changes in in vivo synaptic strength has been a difficult yet essential aim for understanding synaptic information storage. With the diversity of AMPARs now well-established, understanding how this diversity is employed and regulated offers avenues to new view-points on information storage in the brain.

Realizing the central role of AMPARs in LTP, the last ~25 years have provided a wealth of insights into receptor regulation stemming from a multitude of experimental approaches. Sequence diversity in the NTD and CTD of the four core subunits, together with a wide variety of stably associating auxiliary subunits, enables fine control of receptor gating, trafficking and location through transient protein interactions, both in the cytosol and in the synaptic cleft. Yet how these anchor points co-operate under various modes of synaptic activity remains mostly enigmatic. Improved genetic and imaging tools, together with electrophysiology to interrogate the synapse on the one hand, and structural studies of the highly diverse (synapse-specific) AMPARs combined with simulations on the other, are expected to advance this central question in synaptic communication.

## Data Availability

The data referenced in Figure 2 are openly available at https://doi.org/10.1038/s41467-021-25281-4.
